# A 14-year prospective cohort study of type 2 diabetes development in Dutch healthy adults of South Asian origin: risk factors and the association with metabolic syndrome and HOMA-IR

**DOI:** 10.1007/s00592-025-02513-3

**Published:** 2025-05-12

**Authors:** Sebastian B Beckmann, Priyanti Bhawan, Tobias Bruning, T. Martijn Kuijper, Sjaam Jainandunsing

**Affiliations:** 1 Division of Nephrology and Transplantation, Erasmus MC, University Medical Center Rotterdam, Rotterdam, The Netherlands; 2 Department of Internal Medicine, Maasstad Hospital, Rotterdam, The Netherlands; 3 Department of Cardiology, Maasstad Hospital, Rotterdam, The Netherlands; 4 Maasstad Academy, Maasstad Hospital, Rotterdam, The Netherlands; 5 Department of Internal Medicine, Anna Hospital, Geldrop, The Netherlands

**Keywords:** Diabetes, Southeast Asian, Metabolic syndrome, Risk factors

## Abstract

**Objective:**

Type 2 Diabetes (T2D) imposes a disproportionate burden on the South Asian population. Their phenotype is characterized by heightened insulin resistance, even in individuals without overt T2D. Commonly used screening tools underestimate the T2D incidence in this population. The Metabolic syndrome (MetS) and the Homeostatic Model Assessment for Insulin Resistance (HOMA-IR) are indicators of insulin resistance; however, their predictive value for the development of T2D remains unexplored.

**Methods:**

Among 698 initially enrolled healthy South Asian adults aged 30 to 65 in a Rotterdam-based cardiovascular disease prevention study, 270 participants were included after a 14-year follow-up. At baseline, an extensive history, physical examination, and metabolic screening were taken. A follow-up assessment of incident T2D was conducted. Multivariable logistic regression models calculated odds ratios (ORs) for MetS, its components, and HOMA-IR and adjusted for confounders.

**Results:**

33 (12.2%) of participants developed T2D. The presence of MetS at baseline showed an adjusted OR of 2.6, (95% confidence interval (CI) 1.2–5.7, p = 0.02) for incident T2D. Fasting plasma glucose was the most strongly associated component of MetS (OR 3.0, CI 1.1–8.6, p = 0.04) HOMA-IR was also associated and showed an OR of 1.2 per point increase (CI 1.0–1.4, p=0.05).

**Conclusions:**

MetS and FPG were the most important predictors of T2D development in this South Asian cohort. These results underscore the value of diverse variables in T2D detection and give insight into which screening tools for T2D prediction should be used in this high-risk population.

**Supplementary Information:**

The online version contains supplementary material available at 10.1007/s00592-025-02513-3.

## Introduction

Among South Asians, there is an exceptionally high and growing prevalence of type 2 diabetes (T2D) [1]. South Asians with T2D have a higher disease-associated mortality rate and more diabetes-related complications, including cardiovascular disease (CVD), than other ethnicities [2]. Several factors contribute to the increased risk and younger age of onset of T2D in this population. These include an increased level of insulin resistance [3] accompanying a decrease in β-cell function [4], even in subjects without overt T2D [5]. Differences in body fat distribution lead to the thin-fat phenotype with increased abdominal fat even at lower body mass index (BMI). This results in a higher waist circumference (WC) [3], leading to hormonal changes, like in adiponectin and leptin [6, 7], and increased inflammation. Diet [8] and reduced physical activity [9] of South Asians seem to play a role as well.

Validated risk scores are endorsed by the American Diabetes Association (ADA) [10] and other national diabetes organizations, including the Dutch Diabetes Federation [11], as a screening tool for T2D. However, these risk scores, such as the AA risk score, were developed and validated primarily in White populations [12] and may underestimate T2D incidence in South Asians [13]. In the Netherlands, which has a substantial South Asian population due to immigration from the former Dutch colony of Suriname in 1975 [14], this has led to a recommendation for triennial fasting plasma glucose (FPG) testing in individuals of South Asian descent starting at age 35, rather than relying on risk scores [15]. To optimize this approach and refine screening strategies in this population, identifying risk factors for incident T2D is essential.

Assessing insulin resistance in non-diabetic South Asians may help identify individuals at high risk for T2D. The clinical syndrome most commonly associated with insulin resistance is metabolic syndrome (MetS) and its components [16]. The presence of MetS might, therefore, aid the prediction of T2D development [17].

Different definitions exist, and they include an elevation in waist circumference (a measure for excess abdominal fat), blood pressure, FPG and triglycerides and reduced high density lipoprotein (HDL)-cholesterol. The International Diabetes Federation’s (IDF) [19] definition of MetS defines a race-specific cut-off for South Asians, paying to the phenotype mentioned previously, and we use this definition in our study.

Additionally, the Homeostatic Model Assessment for Insulin Resistance (HOMA-IR) offers an alternative to biochemically quantify insulin resistance as an index of FPG and fasting insuling [20].

While cross-sectional studies have explored MetS in the South Asian population, prospective investigations are scarce [21–24]. Given the array of MetS definitions and controversies surrounding its clinical relevance, elucidating its predictive capacity and constituent factors in this demographic is paramount. While an association of HOMA-IR with MetS in South Asians has been shown before [25], its implications for the development of T2D remain unclear.

With this study, we explore the association between IDF MetS, its components, and HOMA-IR with the development of T2D in the adult South Asian population in the Netherlands. Consecutively, we seek to advance the understanding of T2D predictors within this community, offering valuable insights for early detection and tailored preventive healthcare interventions.

## Methodology

### Participants, testing, and follow-up

We employed a prospective longitudinal cohort design. Between 2008 and 2010, 698 participants meeting the inclusion criteria outlined below were enrolled at the Medical Center Rijnmond South (now Maasstad Hospital), a secondary teaching hospital in Rotterdam, the Netherlands. The follow-up reported in this study was conducted in 2023.

Participants were included if they self-identified as South Asian, having at least one ancestor from Surinam, India, Pakistan, or Bangladesh, were healthy (without prior CVD or T2D, and not under treatment for T2D, high blood pressure, high cholesterol, or heart complaints), and were between 30 and 65 years old. Recruitment was facilitated through the Rotterdam Rijnmond Health Municipality and local radio stations with predominantly South Asian listeners, with interested individuals registering via the hospital's cardiovascular department.

Baseline data collection included lifestyle questionnaires covering physical activity, smoking, and alcohol consumption. Participants reported ancestry and family history of T2D and education level. Blood samples were obtained after an overnight fast for lipid markers, glycated hemoglobin (HbA1c), fasting plasma glucose (FPG), and insulin. HOMA-IR was calculated using the formula fasting insulin (mIU/L) × FPG (mmol/L)/22.5. Subsequent in-person interviews gathered medical history and physical examination data, including weight, length, blood pressure, and waist and hip circumference.

During the 2023 follow-up, participants were contacted by phone to inquire about cardiovascular events and T2D occurrence. A standardized questionnaire was used. Permission was sought to retrieve further details from general practitioners or medical records. In cases of death, attempts were made to ascertain CVD presence and cause of death. A maximum of three attempts were made to reach participants by phone.

### Ethical approval

The protocol for the trial was approved by the medical ethical committee (CCMO register number NL24276.101.09). The participants provided written informed consent. The Medical Centre Rijnmond-South funded the initiative from healthcare innovation project budgets by health insurers.

### Statistical analysis

The collected data was analyzed using Stata 15.1. Participants meeting inclusion criteria, with no missing data for relevant variables and completing follow-up, were included in the primary analysis. Baseline characteristics were summarized and compared between groups by chi-squared tests for categorical data and independent t-tests for continuous variables. The skewness of continuous variables was visually assessed by drawing histograms and density plots (Supplemental Fig. 1). Baseline characteristics are presented as a mean for normally distributed continuous variables, as a geometric mean for skewed variables, and as a percentage for categorical data. Logistic regression assessed the univariate and adjusted associations between MetS and its components as dichtomized, HOMA-IR as continuous variable, and incident T2D at follow-up; adjustments ere made for the following potential confounders: sex, age, body mass index (BMI), smoking, education, positive family history of T2D, and physical activity. The set of covariates required for adjustment was substantiated by constructing a directed acyclic graph (DAG) (Supplementary Fig. 1) [26]. Associations in the DAG were drawn liberally and based on common clinical knowledge and reasoning.

The potential impact of unmeasured confounding was assessed by the E-value method [27]. In addition, sensitivity analyses were performed to evaluate the effect of including MetS components as continuous covariates and potential effect modification of MetS by sex and family history of T2D.

Furthermore, to assess the effects of missing data due to participants lost to follow-up, we conducted multiple imputation by chained equations, creating m = 100 imputation datasets and repeated the aforementioned regression analyses. Details of the multiple imputation model are provided in Supplemental Table 3 and Supplemental Data 2.

Statistical significance was assumed at p < 0.05.

## Results

**Fig. 1:**
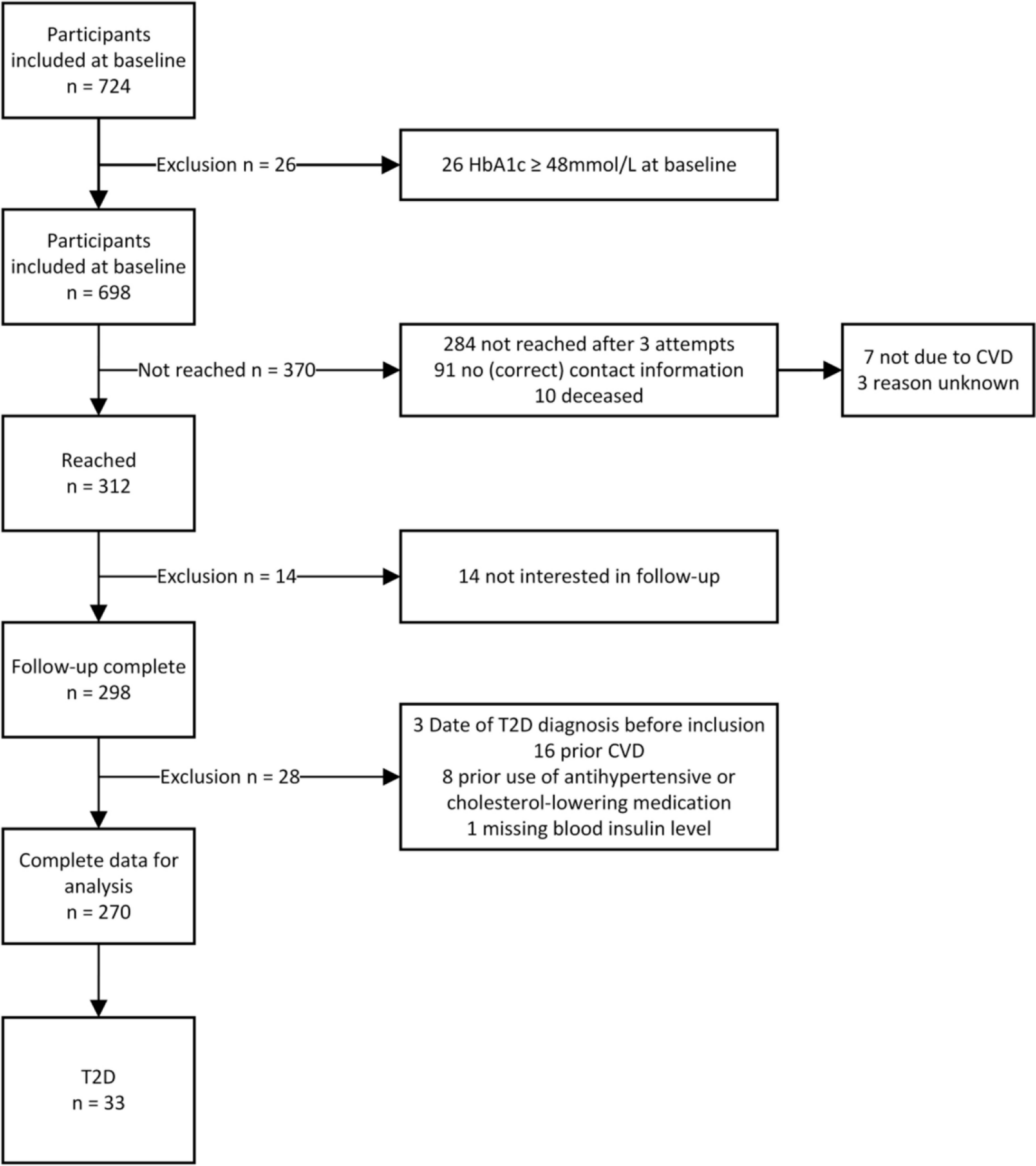
Flowchart of inclusion and exclusion process. CVD: cardiovascular disease, T2D: Type 2 Diabetes

The average follow-up time was 14 years, ranging from 12 to 15 years. Of the 698 participants that did not show biochemical signs of T2D at baseline defined as HbA1c ≥ 48 mmol/L, 312 participants (45%) were reached during the follow-up period, and 298 provided data. After excluding 28 subjects who reported at follow-up that they did not meet the inclusion criteria at baseline and one subject with missing lab results, the final analysis included 270 participants. Among them, 33 individuals (12.2%) self-reported the new onset of T2D. The flowchart (Fig. 1) provides an overview of the in- and exclusion process and the follow-up procedures.

Small differences existed between the participants reached during the follow-up and those not. This was a higher age and higher FPG in the group not reached (46 vs 44 years, 5.0 vs 4.9 mmol/L; see Supplementary Table 1).

Among the 33 participants who developed T2D after inclusion, 28 could provide an estimated date of diagnosis, though 11 noted to be unsure about the exact year of diagnosis. Based on the participants’ estimates the age at diagnosis ranged from 36 to 71 years, averaging 51.5 (standard deviation (SD) 8.8) years. Table 1 presents the baseline characteristics of those who developed T2D compared to those who did not. Notably, 11% (n = 17) of females and 15% (n = 16) of males developed T2D. 88 participants (33%) fulfilled the IDF criteria for MetS at baseline, of whom 19 (22%) developed T2D, while only 14 (8%) of individuals without MetS developed T2D. Differences in MetS prevalence were observed between sexes, with higher rates among males. However, age and sex distribution were similar between groups, while significant differences were noted in positive T2D family history, BMI, WC, HbA1c, insulin, triglycerides, FPG, and HOMA-IR (Supplemental Table 2) (see Table 2).

**Table 1:** Baseline data per group

Characteristic	Total (n = 270)	No T2D at follow-up (n = 237)	Incident T2D during follow-up (n = 33)	p-value
Sex—female	160 (59%)	143 (60%)	17 (52%)	0.33
Age (years)	44 (7)	44 (7)	44(7)	0.88
MetS IDF	88 (33%)	69 (29%)	19 (58%)	0.001
Highest level of education				
Low/Middle	214 (79%)	186 (78%)	28 (85%)	0.40
High	56 (21%)	51 (22%)	5 (15%)	
Smoking	47 (17%)	41 (17%)	6 (18%)	0.90
Positive family history of T2D	222 (82%)	191 (81%)	31 (94%)	0.06
Sport < 3 days per week	164 (61%)	145 (61%)	19 (58%)	0.69
Systolic blood pressure (mmHg)	132 (15)	132 (15)	136 (16)	0.19
Diastolic blood pressure (mmHg)	85 (9)	85 (9)	87 (10)	0.29
HbA1c (mmol/mol)	38 (4)	37 (3)	41 (4)	< 0.001
LDL-C (mmol/L)	3.4 (0.8)	3.4 (0.8)	3.6 (1.1)	0.30
HDL-C (mmol/L)	1.3 (0.3)	1.3 (0.3)	1.2 (0.3)	0.18
Triglycerides (mmol/L)	1.1 (×/1.7)	1.1 (×/1.7)	1.4 (×/1.5)	0.016
Insulin (mIU/L)	10.4 (×/2.2)	9.8 (×/2.2)	15.5 (×/2.0)	0.002
Glucose (mmol/L)	4.9 (0.5)	4.9 (0.4)	5.1 (0.6)	0.008
HOMA-IR	2.3 (×/2.2)	2.1 (×/2.2)	3.5 (×/1.9)	< 0.001
Waist circumference (cm)	89 (10)	89 (10)	93 (9)	0.017
Waist/hip ratio	0.88 (0.07)	0.88 (0.07)	0.9 (0.09)	0.097
BMI (kg/m^2^)	26.4 (4.0)	26.1 (3.9)	28.2 (4.5)	0.007

Data are presented as mean (SD) or geometric mean (× /geometric SD) for continuous measures and % for categorical data

*BMI* body mass index, *CI* confidence interval, *FPG* fasting plasma glucose, *HbA1c* glycated hemoglobin A1c, *HDL-C* high-density lipoprotein cholesterol, *HOMA-IR* Homeostatic Model Assessment for Insulin Resistance, *IDF* International Diabetes Federation, *LDL-C* low-density lipoprotein cholesterol, *MetS* metabolic syndrome, *T2D* type 2 diabetes, *WC* waist circumference

**Table 2:** Univariate and multivariate logistic regression analysis of metabolic syndrome and its components used confounders and HOMA-IR with T2D

Parameter	OR (95% CI)	p-value	OR (95% CI)	p-value
	Univariate analysis		Multivariable analysis	
Metabolic Syndrome				
Metabolic syndrome (IDF)	**3.3 (1.6–7.0)**	** < 0.01**	**2.6 (1.2–5.7)**^§^	**0.02**
Confounders				
Male sex	1.4 (0.7–3.0)	0.34	1.4 (0.6–3.0)	0.41
Age	1.0 (1.0–1.0)	0.95	1.0 (0.9–1.1)	0.98
Current smoking	1.0 (0.4–2.7)	0.90	1.0 (0.6–2.7)	0.96
High education	0.7 (0.2–1.7)	0.40	0.7 (0.2–1.9)	0.44
Positive family history of T2D	3.7 (0.9–16.2)	0.08	3.4 (0.8–15.1)	0.11
Sport ≥ 3 days per week	1.2 (0.6–2.4)	0.69	1.3 (0.6–2.8)	0.54
BMI (per point increase)	**1.1 (1.0–1.2)**	** < 0.01**	1.1 (1.0–1.2)	0.07
Components MetS				
Systolic blood pressure > 129 mmHg/Diastolic blood pressure > 84 mmHg	1.4 (0.6–3.1)	0.39	1.0 (0.5–2.5)^§^	0.89^§^
HDL-C < 1.0 (male)/ < 1.3 (female) mmol/L	1.8 (0.9–3.0)	0.12	1.6 (0.7–3.6)^§^	0.23^§^
FPG 5.6—6.9 mmol/L	**3.2 (1.3–8.5)**	**0.02**	**3.0 (1.1–8.6)**^§^	**0.04**^§^
Triglycerides > 1.7 mmol/L	2.0 (0.9–4.6)	0.07	1.7 (0.7–4.0)^§^	0.23^§^
Waist circumference (IDF) > 89 cm (male)/ > 79 cm (female)	2.6 (1.0–7.0)	0.059	1.7 (0.6–5.2)^§^	0.35^§^
HOMA-IR				
HOMA-IR (per point increase)	**1.3 (1.1–1.5)**	**0.02**	**1.2 (1.0–1.4)**^§^	**0.05**^§^

Multivariate analysis was corrected for sex, age, level of education, physical activity, BMI, smoking, and family history of T2D

*BMI* body mass index, *CI* confidence interval, *FPG* fasting plasma glucose, *HDL-C* high-density lipoprotein cholesterol, *HOMA-IR* Homeostatic Model Assessment for Insulin Resistance, *IDF* International Diabetes Federation, *LDL-C* low-density lipoprotein cholesterol, *OR* odds ratio, *WC* waist circumference

^§^Adjusted for sex, age, smoking, education, family history of T2D, physical activity (categorical), and BMI (coefficients not shown)

In the unadjusted logistic regression model, MetS at baseline, according to IDF criteria, exhibited an odds ratio (OR) of 3.3 [95% CI 1.6–7.0, p < 0.01], corresponding to a 24% predicted probability of developing T2D within 15 years. Each point increase in HOMA-IR was associated with a significantly raised probability of developing T2D with an OR of 1.3 [95% CI 1.1–1.5, p = 0.02]. Among individual MetS criteria, elevated FPG showed a significant association with the development of T2D. Numerically, the largest association with an OR of 3.7 [95% CI 0.9–16.2, p = 0.08] was found for a positive family history of T2D, which did not reach the prespecified level of significance. In the unadjusted model, BMI was associated with the development of T2D as well [OR 1.1, 95% CI 1.0–1.2, p < 0.01]

Adjusting for potential confounders (see the DAG in Supplementary Fig. 1) in the second model slightly attenuated the associations, but they remained significant for both MetS and HOMA-IR. Specifically, according to IDF criteria, MetS had an OR of 2.6 [95% CI 1.2–5.7, p = 0.02]. BMI did not show a significant association anymore. In the adjusted analyses, a positive family history of T2D again demonstrated the highest OR of 3.4 [95% CI 0.8–15.1, p = 0.11] but missed the significance level. We also performed the regression analysis for previously dichotomized variables as continuous, which did not change our results (Supplemental Data 3).

The potential impact of unmeasured confounding was assessed by using the E-value method [27]. For the adjusted OR of MetS [OR = 2.6, 95% CI 1.2–5.7], the corresponding E-value was 4.6 [95% CI 1.8–10.9]. This means that a (set of) potential unmeasured confounder(s) needs to have an association of at least 4.6 with either the exposure or the outcome to explain away the found association.

To assess the potential impact of patients lost to follow-up, multiple imputation was performed, which did not change our findings (Supplemental Data 2).

## Discussion

Our study reveals significant positive associations between the presence of MetS and the development of T2D in a healthy Dutch cohort of South Asian descent, both unadjusted and after adjustment for confounders. The FPG criterion (5.6–6.9 mmol/L) emerged as the most strongly associated individual component of MetS. This observation corroborates findings from prior studies, emphasizing the predictive value of elevated FPG levels in various populations [28]. Furthermore, HOMA-IR was positively associated with T2D development, underscoring its potential utility in risk assessment.

The observed incidence of T2D (12.2%) aligns with previous findings [29], highlighting the substantial disease burden among the South Asian population in the Netherlands. We could demonstrate that those showing physical or biochemical signs of insulin resistance as expressed by the presence of MetS are at a high risk of developing T2D later in their lives despite being classified as free of disease at baseline.

The present study forms the largest prospective cohort of South Asians in the Netherlands reported in the literature. The longitudinal design and follow-up of 14 years make our study stand out. However, the results of our study are limited by the small sample size due to the high percentage of participants not reached at follow-up, even after three attempts. We tried to mitigate the effects of the participants lost to follow-up by performing multiple imputation. This did not change our results. However, interpreting the results of multiple imputation can become more challenging the more data is missing. Comparing the baseline characteristics of the group reached and not reached revealed a small but statistically significant difference in FPG and age, with higher values in the group not reached (46 vs. 44 years, 5.0 vs. 4.9 mmol/L, Supplementary Table 1). As age and FPG are related to the development of T2D, a higher rate of incident T2D is expected in the group not reached, leading to a potential underestimation of the effect of MetS on T2D development in our analysis. Based on the pathophysiology, waist circumference and elevated triglycerides would be expected to also show an association with T2D in a sample of larger size.

Another limitation of our study is the potential selection bias due to the recruitment strategy relying on interested individuals to contact the study center themselves. It is possible that predominantly individuals with a perceived higher risk of T2D followed the invitation to participate in the study, as the “reward” for participation was a complete physical and laboratory check. This can lead to an overestimation of the incidence of T2D compared to the general South Asian population in the Netherlands. This could also explain why we were unable to demonstrate a significant association between a positive family history of and incident T2D, as 82% of the whole population had a positive family history. Nonetheless, the high OR for a positive family history of T2D suggests a strong genetic predisposition, consistent with the established pathophysiology of the disease [30].

The preferred model for studying associations between risk factors and incident disease would be a time-to-event analysis. We chose not to perform such a model due to two reasons: (1) there is usually a relevant delay between the moment of onset of T2D and its clinical diagnosis [31], and (2) participants were often unable to provide an exact date of diagnosis during the follow-up interview. Future studies should incorporate more frequent follow-up assessments to mitigate this limitation.

Overall, classic risk factors for T2D affected participants with MetS more than those without (Supplementary Table 2). The low or absent degree of association of factors other than BMI and FPG with the development of T2D underlines the diverse pathophysiology of MetS once more.

Our study affirms the association of HOMA-IR with T2D development in this population, suggesting preferential use for risk assessment [25]. However, the accuracy of HOMA-IR on an individual level remains debatable due to inherent measurement variances and a lack of standardized cut-off values [32]. Based on our results, relying on an elevated FPG level as a screening tool seems appropriate, which is the current standard in this population in the Netherlands [15].

One advantage of studying South Asians in the Netherlands is that this group is uniform regarding exposure to Western lifestyle and diet. The majority of this population migrated to the Netherlands around 1975, following Suriname’s independence [14]. Consequently, we did not adjust for the duration of residence in the Netherlands. When comparing our findings to studies on risk factors for T2D in South Asians living in South Asia, the potential impact of Western lifestyle and dietary exposure should be taken into account [9, 21]. However, the prevalence of MetS in our cohort was largely the same as in cohorts in urban South Asia [21] and the United States. [22]

Our findings hold practical implications for targeted interventions among the South Asian population, emphasizing the importance of early risk assessment and tailored preventive strategies. In particular, South Asians with elevated FPG require close monitoring for signs of T2D. While these findings do not immediately warrant immediate guideline changes, they underscore the need for continued research to refine T2D risk prediction models.

## Supplementary Information

Below is the link to the electronic supplementary material.

Supplementary Data 1 (DOCX 54 KB)

Supplementary Data 2 (DOCX 381 KB)

Supplementary Data 3 (DOCX 47 KB)

Supplementary Data 4 (DOCX 19 KB)

Supplementary Figure 1 (DOCX 131 KB)

## Abbreviations

BMI: Body mass index
CI: Confidence Interval
CVD: Cardiovascular disease
DAG: Causal Directed Acyclic Graph
HbA1c: Glycated hemoglobin A1c
HOMA-IR: Homeostatic Model Assessment for Insulin Resistance
FPG: Fasting plasma glucose
HDL-C: High-density lipoprotein cholesterol
IDF: International Diabetes Federation
LDL-C: Low-density lipoprotein cholesterol
MetS: Metabolic Syndrome
OR: Odds ratio
T2D: Type 2 diabetes
WC: Waist circumference
