# Supplementary material for: A 14-year prospective cohort study of type 2 diabetes development in Dutch healthy adults of South Asian origin: risk factors and the association with metabolic syndrome and HOMA-IR
Source: Acta Diabetol. 2025 May 12;62(11):1873–80. doi: 10.1007/s00592-025-02513-3 (PMC12640338; doi:10.1007/s00592-025-02513-3)
Supplement: Supplementary file 1 — Supplementary Data 1 (DOCX 54 KB) [file 592_2025_2513_MOESM1_ESM.docx]

**Supplemental Data 1 - Univariate and multivariable analyses**

**Univariate analyses**

Logistic regression

| T2D | OR | | St.Err. | z-value | | p-value | [95% Conf | | Interval] | | Sig |
| --- | --- | --- | --- | --- | --- | --- | --- | --- | --- | --- | --- |
| MetS (IDF) | 3.304 | | 1.256 | 3.14 | | .002 | 1.569 | | 6.961 | | *** |
| Constant | .083 | | .023 | -8.93 | | 0 | .048 | | .144 | | *** |
|  | | | | | | | | | | | |
| Mean dependent var | | 0.122 | | | SD dependent var | | | 0.328 | |  |  |
| Pseudo r-squared | | 0.050 | | | Number of obs | | | 270 | |  |  |
| Chi-square | | 9.989 | | | Prob > chi2 | | | 0.002 | |  |  |
| Akaike crit. (AIC) | | 194.529 | | | Bayesian crit. (BIC) | | | 201.726 | |  |  |
| **** p<.01, ** p<.05, * p<.1* | | | | | | | | | | | |
|  | | | | | | | | | | | |

Logistic regression

| T2D | OR | | St.Err. | z-value | | p-value | [95% Conf | | Interval] | | Sig |
| --- | --- | --- | --- | --- | --- | --- | --- | --- | --- | --- | --- |
| Sex (male) | 1.432 | | .534 | 0.96 | | .336 | .69 | | 2.973 | |  |
| Constant | .119 | | .03 | -8.30 | | 0 | .072 | | .197 | | *** |
|  | | | | | | | | | | | |
| Mean dependent var | | 0.122 | | | SD dependent var | | | 0.328 | |  |  |
| Pseudo r-squared | | 0.005 | | | Number of obs | | | 270 | |  |  |
| Chi-square | | 0.922 | | | Prob > chi2 | | | 0.337 | |  |  |
| Akaike crit. (AIC) | | 203.596 | | | Bayesian crit. (BIC) | | | 210.793 | |  |  |
| **** p<.01, ** p<.05, * p<.1* | | | | | | | | | | | |
|  | | | | | | | | | | | |

Logistic regression

| T2D | OR | | St.Err. | z-value | | p-value | [95% Conf | | Interval] | | Sig |
| --- | --- | --- | --- | --- | --- | --- | --- | --- | --- | --- | --- |
| Age (years) | 1.002 | | .027 | 0.06 | | .952 | .951 | | 1.055 | |  |
| Constant | .13 | | .155 | -1.71 | | .088 | .012 | | 1.355 | | * |
|  | | | | | | | | | | | |
| Mean dependent var | | 0.122 | | | SD dependent var | | | 0.328 | |  |  |
| Pseudo r-squared | | 0.000 | | | Number of obs | | | 270 | |  |  |
| Chi-square | | 0.004 | | | Prob > chi2 | | | 0.952 | |  |  |
| Akaike crit. (AIC) | | 204.514 | | | Bayesian crit. (BIC) | | | 211.711 | |  |  |
| **** p<.01, ** p<.05, * p<.1* | | | | | | | | | | | |
|  | | | | | | | | | | | |

Logistic regression

| T2D | OR | | St.Err. | z-value | | p-value | [95% Conf | | Interval] | | Sig |
| --- | --- | --- | --- | --- | --- | --- | --- | --- | --- | --- | --- |
| Current smoking | 1.062 | | .513 | 0.13 | | .9 | .412 | | 2.737 | |  |
| Constant | .138 | | .028 | -9.66 | | 0 | .092 | | .206 | | *** |
|  | | | | | | | | | | | |
| Mean dependent var | | 0.122 | | | SD dependent var | | | 0.328 | |  |  |
| Pseudo r-squared | | 0.000 | | | Number of obs | | | 270 | |  |  |
| Chi-square | | 0.016 | | | Prob > chi2 | | | 0.901 | |  |  |
| Akaike crit. (AIC) | | 204.502 | | | Bayesian crit. (BIC) | | | 211.699 | |  |  |
| **** p<.01, ** p<.05, * p<.1* | | | | | | | | | | | |
|  | | | | | | | | | | | |

Logistic regression

| T2D | OR | | St.Err. | z-value | | p-value | [95% Conf | | Interval] | | Sig |
| --- | --- | --- | --- | --- | --- | --- | --- | --- | --- | --- | --- |
| Education (high) | .651 | | .333 | -0.84 | | .401 | .239 | | 1.772 | |  |
| Constant | .151 | | .031 | -9.34 | | 0 | .101 | | .224 | | *** |
|  | | | | | | | | | | | |
| Mean dependent var | | 0.122 | | | SD dependent var | | | 0.328 | |  |  |
| Pseudo r-squared | | 0.004 | | | Number of obs | | | 270 | |  |  |
| Chi-square | | 0.763 | | | Prob > chi2 | | | 0.383 | |  |  |
| Akaike crit. (AIC) | | 203.755 | | | Bayesian crit. (BIC) | | | 210.952 | |  |  |
| **** p<.01, ** p<.05, * p<.1* | | | | | | | | | | | |
|  | | | | | | | | | | | |

Logistic regression

| T2D | OR | | St.Err. | z-value | | p-value | [95% Conf | | Interval] | | Sig |
| --- | --- | --- | --- | --- | --- | --- | --- | --- | --- | --- | --- |
| Family history of T2D | 3.733 | | 2.792 | 1.76 | | .078 | .862 | | 16.166 | | * |
| Constant | .043 | | .031 | -4.34 | | 0 | .011 | | .179 | | *** |
|  | | | | | | | | | | | |
| Mean dependent var | | 0.122 | | | SD dependent var | | | 0.328 | |  |  |
| Pseudo r-squared | | 0.022 | | | Number of obs | | | 270 | |  |  |
| Chi-square | | 4.377 | | | Prob > chi2 | | | 0.036 | |  |  |
| Akaike crit. (AIC) | | 200.141 | | | Bayesian crit. (BIC) | | | 207.338 | |  |  |
| **** p<.01, ** p<.05, * p<.1* | | | | | | | | | | | |
|  | | | | | | | | | | | |

Logistic regression

| T2D | OR | | St.Err. | z-value | | p-value | [95% Conf | | Interval] | | Sig |
| --- | --- | --- | --- | --- | --- | --- | --- | --- | --- | --- | --- |
| Sport (>2x/week) | 1.161 | | .437 | 0.40 | | .691 | .555 | | 2.429 | |  |
| Constant | .131 | | .032 | -8.33 | | 0 | .081 | | .211 | | *** |
|  | | | | | | | | | | | |
| Mean dependent var | | 0.122 | | | SD dependent var | | | 0.328 | |  |  |
| Pseudo r-squared | | 0.001 | | | Number of obs | | | 270 | |  |  |
| Chi-square | | 0.157 | | | Prob > chi2 | | | 0.692 | |  |  |
| Akaike crit. (AIC) | | 204.361 | | | Bayesian crit. (BIC) | | | 211.558 | |  |  |
| **** p<.01, ** p<.05, * p<.1* | | | | | | | | | | | |
|  | | | | | | | | | | | |

Logistic regression

| T2D | OR | | St.Err. | z-value | | p-value | [95% Conf | | Interval] | | Sig |
| --- | --- | --- | --- | --- | --- | --- | --- | --- | --- | --- | --- |
| BMI (kg/m^2^) | 1.112 | | .046 | 2.60 | | .009 | 1.027 | | 1.205 | | *** |
| Constant | .008 | | .009 | -4.22 | | 0 | .001 | | .074 | | *** |
|  | | | | | | | | | | | |
| Mean dependent var | | 0.122 | | | SD dependent var | | | 0.328 | |  |  |
| Pseudo r-squared | | 0.032 | | | Number of obs | | | 270 | |  |  |
| Chi-square | | 6.482 | | | Prob > chi2 | | | 0.011 | |  |  |
| Akaike crit. (AIC) | | 198.036 | | | Bayesian crit. (BIC) | | | 205.233 | |  |  |
| **** p<.01, ** p<.05, * p<.1* | | | | | | | | | | | |
|  | | | | | | | | | | | |

Logistic regression

| T2D | OR | | St.Err. | z-value | | p-value | [95% Conf | | Interval] | | Sig |
| --- | --- | --- | --- | --- | --- | --- | --- | --- | --- | --- | --- |
| Blood pressure (cat.) | 1.408 | | .566 | 0.85 | | .394 | .641 | | 3.095 | |  |
| Constant | .111 | | .037 | -6.59 | | 0 | .058 | | .214 | | *** |
|  | | | | | | | | | | | |
| Mean dependent var | | 0.122 | | | SD dependent var | | | 0.328 | |  |  |
| Pseudo r-squared | | 0.004 | | | Number of obs | | | 270 | |  |  |
| Chi-square | | 0.750 | | | Prob > chi2 | | | 0.387 | |  |  |
| Akaike crit. (AIC) | | 203.768 | | | Bayesian crit. (BIC) | | | 210.965 | |  |  |
| **** p<.01, ** p<.05, * p<.1* | | | | | | | | | | | |
|  | | | | | | | | | | | |

Logistic regression

| T2D | OR | | St.Err. | z-value | | p-value | [95% Conf | | Interval] | | Sig |
| --- | --- | --- | --- | --- | --- | --- | --- | --- | --- | --- | --- |
| HDL-C (cat.) | 1.779 | | .666 | 1.54 | | .124 | .855 | | 3.704 | |  |
| Constant | .11 | | .028 | -8.65 | | 0 | .066 | | .181 | | *** |
|  | | | | | | | | | | | |
| Mean dependent var | | 0.122 | | | SD dependent var | | | 0.328 | |  |  |
| Pseudo r-squared | | 0.012 | | | Number of obs | | | 270 | |  |  |
| Chi-square | | 2.342 | | | Prob > chi2 | | | 0.126 | |  |  |
| Akaike crit. (AIC) | | 202.176 | | | Bayesian crit. (BIC) | | | 209.373 | |  |  |
| **** p<.01, ** p<.05, * p<.1* | | | | | | | | | | | |
|  | | | | | | | | | | | |

Logistic regression

| T2D | OR | | St.Err. | z-value | | p-value | [95% Conf | | Interval] | | Sig |
| --- | --- | --- | --- | --- | --- | --- | --- | --- | --- | --- | --- |
| FPG (cat.) | 3.276 | | 1.61 | 2.41 | | .016 | 1.25 | | 8.581 | | ** |
| Constant | .119 | | .025 | -10.27 | | 0 | .079 | | .178 | | *** |
|  | | | | | | | | | | | |
| Mean dependent var | | 0.122 | | | SD dependent var | | | 0.328 | |  |  |
| Pseudo r-squared | | 0.025 | | | Number of obs | | | 270 | |  |  |
| Chi-square | | 5.088 | | | Prob > chi2 | | | 0.024 | |  |  |
| Akaike crit. (AIC) | | 199.430 | | | Bayesian crit. (BIC) | | | 206.627 | |  |  |
| **** p<.01, ** p<.05, * p<.1* | | | | | | | | | | | |
|  | | | | | | | | | | | |

Logistic regression

| T2D | OR | | St.Err. | z-value | | p-value | [95% Conf | | Interval] | | Sig |
| --- | --- | --- | --- | --- | --- | --- | --- | --- | --- | --- | --- |
| Triglycerides (cat.) | 2.076 | | .839 | 1.81 | | .071 | .94 | | 4.584 | | * |
| Constant | .115 | | .026 | -9.60 | | 0 | .074 | | .179 | | *** |
|  | | | | | | | | | | | |
| Mean dependent var | | 0.122 | | | SD dependent var | | | 0.328 | |  |  |
| Pseudo r-squared | | 0.015 | | | Number of obs | | | 270 | |  |  |
| Chi-square | | 3.063 | | | Prob > chi2 | | | 0.080 | |  |  |
| Akaike crit. (AIC) | | 201.455 | | | Bayesian crit. (BIC) | | | 208.652 | |  |  |
| **** p<.01, ** p<.05, * p<.1* | | | | | | | | | | | |
|  | | | | | | | | | | | |

Logistic regression

| T2D | OR | | St.Err. | z-value | | p-value | [95% Conf | | Interval] | | Sig |
| --- | --- | --- | --- | --- | --- | --- | --- | --- | --- | --- | --- |
| Waist circumference (cat.) | 2.593 | | 1.31 | 1.89 | | .059 | .963 | | 6.978 | | * |
| Constant | .067 | | .031 | -5.86 | | 0 | .027 | | .165 | | *** |
|  | | | | | | | | | | | |
| Mean dependent var | | 0.122 | | | SD dependent var | | | 0.328 | |  |  |
| Pseudo r-squared | | 0.021 | | | Number of obs | | | 270 | |  |  |
| Chi-square | | 4.227 | | | Prob > chi2 | | | 0.040 | |  |  |
| Akaike crit. (AIC) | | 200.291 | | | Bayesian crit. (BIC) | | | 207.488 | |  |  |
| **** p<.01, ** p<.05, * p<.1* | | | | | | | | | | | |
|  | | | | | | | | | | | |

Logistic regression

| T2D | OR | | St.Err. | z-value | | p-value | [95% Conf | | Interval] | | Sig |
| --- | --- | --- | --- | --- | --- | --- | --- | --- | --- | --- | --- |
| HOMA-IR | 1.273 | | .099 | 3.10 | | .002 | 1.093 | | 1.483 | | *** |
| Constant | .062 | | .021 | -8.06 | | 0 | .031 | | .122 | | *** |
|  | | | | | | | | | | | |
| Mean dependent var | | 0.122 | | | SD dependent var | | | 0.328 | |  |  |
| Pseudo r-squared | | 0.050 | | | Number of obs | | | 270 | |  |  |
| Chi-square | | 9.977 | | | Prob > chi2 | | | 0.002 | |  |  |
| Akaike crit. (AIC) | | 194.541 | | | Bayesian crit. (BIC) | | | 201.738 | |  |  |
| **** p<.01, ** p<.05, * p<.1* | | | | | | | | | | | |
|  | | | | | | | | | | | |

**Multivariable analyses**

Logistic regression

| T2D | OR | | St.Err. | z-value | | p-value | [95% Conf | | Interval] | | Sig |
| --- | --- | --- | --- | --- | --- | --- | --- | --- | --- | --- | --- |
| MetS (IDF) | 2.588 | | 1.051 | 2.34 | | .019 | 1.167 | | 5.737 | | ** |
| Age (years) | .999 | | .029 | -0.03 | | .979 | .944 | | 1.058 | |  |
| Sex (male) | 1.382 | | .548 | 0.82 | | .415 | .635 | | 3.005 | |  |
| Education (high) | .665 | | .353 | -0.77 | | .442 | .235 | | 1.88 | |  |
| Sport (>2x/week) | 1.28 | | .511 | 0.62 | | .536 | .586 | | 2.799 | |  |
| BMI (kg/m^2^) | 1.085 | | .049 | 1.80 | | .072 | .993 | | 1.186 | | * |
| Current smoking | 1.025 | | .525 | 0.05 | | .962 | .375 | | 2.799 | |  |
| Family history of T2D | 3.371 | | 2.581 | 1.59 | | .112 | .752 | | 15.114 | |  |
| Constant | .003 | | .006 | -2.97 | | .003 | 0 | | .139 | | *** |
|  | | | | | | | | | | | |
| Mean dependent var | | 0.122 | | | SD dependent var | | | 0.328 | |  |  |
| Pseudo r-squared | | 0.090 | | | Number of obs | | | 270 | |  |  |
| Chi-square | | 18.050 | | | Prob > chi2 | | | 0.021 | |  |  |
| Akaike crit. (AIC) | | 200.468 | | | Bayesian crit. (BIC) | | | 232.854 | |  |  |
| **** p<.01, ** p<.05, * p<.1* | | | | | | | | | | | |
|  | | | | | | | | | | | |

Logistic regression

| T2D | OR | | St.Err. | z-value | | p-value | [95% Conf | | Interval] | | Sig |
| --- | --- | --- | --- | --- | --- | --- | --- | --- | --- | --- | --- |
| Blood pressure (cat.) | 1.06 | | .455 | 0.14 | | .892 | .457 | | 2.458 | |  |
| Age (years) | .995 | | .028 | -0.20 | | .845 | .941 | | 1.051 | |  |
| Sex (male) | 1.565 | | .608 | 1.15 | | .249 | .731 | | 3.352 | |  |
| Education (high) | .68 | | .358 | -0.73 | | .464 | .243 | | 1.907 | |  |
| Sport (>2x/week) | 1.162 | | .452 | 0.38 | | .701 | .541 | | 2.492 | |  |
| BMI (kg/m^2^) | 1.113 | | .048 | 2.47 | | .014 | 1.022 | | 1.211 | | ** |
| Current smoking | 1.171 | | .587 | 0.31 | | .753 | .438 | | 3.13 | |  |
| Family history of T2D | 3.654 | | 2.79 | 1.70 | | .09 | .818 | | 16.316 | | * |
| Constant | .002 | | .005 | -3.18 | | .001 | 0 | | .099 | | *** |
|  | | | | | | | | | | | |
| Mean dependent var | | 0.122 | | | SD dependent var | | | 0.328 | |  |  |
| Pseudo r-squared | | 0.063 | | | Number of obs | | | 270 | |  |  |
| Chi-square | | 12.548 | | | Prob > chi2 | | | 0.128 | |  |  |
| Akaike crit. (AIC) | | 205.970 | | | Bayesian crit. (BIC) | | | 238.356 | |  |  |
| **** p<.01, ** p<.05, * p<.1* | | | | | | | | | | | |
|  | | | | | | | | | | | |

Logistic regression

| T2D | OR | | St.Err. | z-value | | p-value | [95% Conf | | Interval] | | Sig |
| --- | --- | --- | --- | --- | --- | --- | --- | --- | --- | --- | --- |
| HDL-C (cat.) | 1.634 | | .667 | 1.20 | | .229 | .734 | | 3.636 | |  |
| Age (years) | 1.006 | | .03 | 0.20 | | .838 | .95 | | 1.066 | |  |
| Sex (male) | 1.54 | | .598 | 1.11 | | .265 | .72 | | 3.295 | |  |
| Education (high) | .651 | | .343 | -0.82 | | .415 | .232 | | 1.827 | |  |
| Sport (>2x/week) | 1.231 | | .485 | 0.53 | | .598 | .569 | | 2.664 | |  |
| BMI (kg/m^2^) | 1.11 | | .048 | 2.42 | | .016 | 1.02 | | 1.209 | | ** |
| Current smoking | 1.199 | | .603 | 0.36 | | .718 | .448 | | 3.211 | |  |
| Family history of T2D | 3.551 | | 2.718 | 1.66 | | .098 | .792 | | 15.915 | | * |
| Constant | .001 | | .003 | -3.31 | | .001 | 0 | | .067 | | *** |
|  | | | | | | | | | | | |
| Mean dependent var | | 0.122 | | | SD dependent var | | | 0.328 | |  |  |
| Pseudo r-squared | | 0.070 | | | Number of obs | | | 270 | |  |  |
| Chi-square | | 13.967 | | | Prob > chi2 | | | 0.083 | |  |  |
| Akaike crit. (AIC) | | 204.551 | | | Bayesian crit. (BIC) | | | 236.937 | |  |  |
| **** p<.01, ** p<.05, * p<.1* | | | | | | | | | | | |
|  | | | | | | | | | | | |

Logistic regression

| T2D | OR | | St.Err. | z-value | | p-value | [95% Conf | | Interval] | | Sig |
| --- | --- | --- | --- | --- | --- | --- | --- | --- | --- | --- | --- |
| FPG (cat.) | 3.039 | | 1.613 | 2.09 | | .036 | 1.074 | | 8.602 | | ** |
| Age (years) | .986 | | .029 | -0.48 | | .631 | .932 | | 1.044 | |  |
| Sex (male) | 1.443 | | .574 | 0.92 | | .356 | .662 | | 3.146 | |  |
| Education (high) | .63 | | .334 | -0.87 | | .383 | .223 | | 1.78 | |  |
| Sport (>2x/week) | 1.141 | | .451 | 0.33 | | .739 | .526 | | 2.475 | |  |
| BMI (kg/m^2^) | 1.107 | | .048 | 2.36 | | .018 | 1.018 | | 1.205 | | ** |
| Current smoking | .981 | | .509 | -0.04 | | .97 | .355 | | 2.711 | |  |
| Family history of T2D | 3.693 | | 2.827 | 1.71 | | .088 | .824 | | 16.554 | | * |
| Constant | .004 | | .007 | -2.90 | | .004 | 0 | | .164 | | *** |
|  | | | | | | | | | | | |
| Mean dependent var | | 0.122 | | | SD dependent var | | | 0.328 | |  |  |
| Pseudo r-squared | | 0.082 | | | Number of obs | | | 270 | |  |  |
| Chi-square | | 16.540 | | | Prob > chi2 | | | 0.035 | |  |  |
| Akaike crit. (AIC) | | 201.978 | | | Bayesian crit. (BIC) | | | 234.364 | |  |  |
| **** p<.01, ** p<.05, * p<.1* | | | | | | | | | | | |
|  | | | | | | | | | | | |

Logistic regression

| T2D | OR | | St.Err. | z-value | | p-value | [95% Conf | | Interval] | | Sig |
| --- | --- | --- | --- | --- | --- | --- | --- | --- | --- | --- | --- |
| Triglycerides (cat.) | 1.697 | | .747 | 1.20 | | .23 | .715 | | 4.023 | |  |
| Age (years) | .997 | | .028 | -0.09 | | .926 | .943 | | 1.054 | |  |
| Sex (male) | 1.367 | | .557 | 0.77 | | .443 | .615 | | 3.039 | |  |
| Education (high) | .704 | | .37 | -0.67 | | .504 | .251 | | 1.972 | |  |
| Sport (>2x/week) | 1.188 | | .465 | 0.44 | | .66 | .552 | | 2.557 | |  |
| BMI (kg/m^2^) | 1.107 | | .048 | 2.33 | | .02 | 1.016 | | 1.206 | | ** |
| Current smoking | 1.133 | | .573 | 0.25 | | .805 | .42 | | 3.053 | |  |
| Family history of T2D | 3.715 | | 2.833 | 1.72 | | .085 | .833 | | 16.558 | | * |
| Constant | .002 | | .004 | -3.19 | | .001 | 0 | | .096 | | *** |
|  | | | | | | | | | | | |
| Mean dependent var | | 0.122 | | | SD dependent var | | | 0.328 | |  |  |
| Pseudo r-squared | | 0.069 | | | Number of obs | | | 270 | |  |  |
| Chi-square | | 13.916 | | | Prob > chi2 | | | 0.084 | |  |  |
| Akaike crit. (AIC) | | 204.602 | | | Bayesian crit. (BIC) | | | 236.988 | |  |  |
| **** p<.01, ** p<.05, * p<.1* | | | | | | | | | | | |
|  | | | | | | | | | | | |

Logistic regression

| T2D | OR | | St.Err. | z-value | | p-value | [95% Conf | | Interval] | | Sig |
| --- | --- | --- | --- | --- | --- | --- | --- | --- | --- | --- | --- |
| Waist circumference (cat.) | 1.708 | | .974 | 0.94 | | .348 | .558 | | 5.224 | |  |
| Age (years) | .994 | | .028 | -0.21 | | .834 | .942 | | 1.05 | |  |
| Sex (male) | 1.618 | | .629 | 1.24 | | .216 | .755 | | 3.467 | |  |
| Education (high) | .696 | | .365 | -0.69 | | .489 | .249 | | 1.946 | |  |
| Sport (>2x/week) | 1.19 | | .465 | 0.44 | | .657 | .553 | | 2.561 | |  |
| BMI (kg/m^2^) | 1.09 | | .053 | 1.79 | | .073 | .992 | | 1.198 | | * |
| Current smoking | 1.151 | | .58 | 0.28 | | .781 | .428 | | 3.091 | |  |
| Family history of T2D | 3.6 | | 2.746 | 1.68 | | .093 | .807 | | 16.059 | | * |
| Constant | .003 | | .006 | -3.07 | | .002 | 0 | | .121 | | *** |
|  | | | | | | | | | | | |
| Mean dependent var | | 0.122 | | | SD dependent var | | | 0.328 | |  |  |
| Pseudo r-squared | | 0.067 | | | Number of obs | | | 270 | |  |  |
| Chi-square | | 13.458 | | | Prob > chi2 | | | 0.097 | |  |  |
| Akaike crit. (AIC) | | 205.060 | | | Bayesian crit. (BIC) | | | 237.446 | |  |  |
| **** p<.01, ** p<.05, * p<.1* | | | | | | | | | | | |
|  | | | | | | | | | | | |

Logistic regression

| T2D | OR | | St.Err. | z-value | | p-value | [95% Conf | | Interval] | | Sig |
| --- | --- | --- | --- | --- | --- | --- | --- | --- | --- | --- | --- |
| HOMA-IR | 1.189 | | .106 | 1.95 | | .051 | .999 | | 1.416 | | * |
| Age (years) | 1 | | .028 | -0.01 | | .991 | .946 | | 1.057 | |  |
| Sex (male) | 1.576 | | .62 | 1.16 | | .248 | .729 | | 3.406 | |  |
| Education (high) | .701 | | .369 | -0.67 | | .5 | .25 | | 1.966 | |  |
| Sport (>2x/week) | 1.202 | | .475 | 0.46 | | .642 | .554 | | 2.608 | |  |
| BMI (kg/m^2^) | 1.063 | | .052 | 1.23 | | .218 | .965 | | 1.171 | |  |
| Current smoking | 1.087 | | .551 | 0.17 | | .869 | .403 | | 2.935 | |  |
| Family history of T2D | 3.317 | | 2.527 | 1.57 | | .115 | .745 | | 14.764 | |  |
| Constant | .004 | | .008 | -2.86 | | .004 | 0 | | .178 | | *** |
|  | | | | | | | | | | | |
| Mean dependent var | | 0.122 | | | SD dependent var | | | 0.328 | |  |  |
| Pseudo r-squared | | 0.082 | | | Number of obs | | | 270 | |  |  |
| Chi-square | | 16.453 | | | Prob > chi2 | | | 0.036 | |  |  |
| Akaike crit. (AIC) | | 202.065 | | | Bayesian crit. (BIC) | | | 234.451 | |  |  |
| **** p<.01, ** p<.05, * p<.1* | | | | | | | | | | | |
|  | | | | | | | | | | | |
