# Supplementary material for: A 14-year prospective cohort study of type 2 diabetes development in Dutch healthy adults of South Asian origin: risk factors and the association with metabolic syndrome and HOMA-IR
Source: Acta Diabetol. 2025 May 12;62(11):1873–80. doi: 10.1007/s00592-025-02513-3 (PMC12640338; doi:10.1007/s00592-025-02513-3)
Supplement: Supplementary file 2 — Supplementary Data 2 (DOCX 381 KB) [file 592_2025_2513_MOESM2_ESM.docx]

**Supplemental Data 2 - Univariate and multivariable analyses - multiple imputation**

**Univariate analyses**

**Multivariable analyses**
