# Supplementary material for: A 14-year prospective cohort study of type 2 diabetes development in Dutch healthy adults of South Asian origin: risk factors and the association with metabolic syndrome and HOMA-IR
Source: Acta Diabetol. 2025 May 12;62(11):1873–80. doi: 10.1007/s00592-025-02513-3 (PMC12640338; doi:10.1007/s00592-025-02513-3)
Supplement: Supplementary file 3 — Supplementary Data 3 (DOCX 47 KB) [file 592_2025_2513_MOESM3_ESM.docx]

**Supplemental Data 3 - Sensitivity analyses**

**MetS components as continuous covariate**

**Univariate analyses**

Logistic regression

| T2D | OR | | St.Err. | z-value | | p-value | [95% Conf | | Interval] | | Sig |
| --- | --- | --- | --- | --- | --- | --- | --- | --- | --- | --- | --- |
| Blood pressure (mmHg) | 1.016 | | .012 | 1.31 | | .189 | .992 | | 1.041 | |  |
| Constant | .016 | | .027 | -2.48 | | .013 | .001 | | .421 | | ** |
|  | | | | | | | | | | | |
| Mean dependent var | | 0.122 | | | SD dependent var | | | 0.328 | |  |  |
| Pseudo r-squared | | 0.008 | | | Number of obs | | | 270 | |  |  |
| Chi-square | | 1.696 | | | Prob > chi2 | | | 0.193 | |  |  |
| Akaike crit. (AIC) | | 202.822 | | | Bayesian crit. (BIC) | | | 210.019 | |  |  |
| **** p<.01, ** p<.05, * p<.1* | | | | | | | | | | | |
|  | | | | | | | | | | | |

Logistic regression

| T2D | OR | | St.Err. | z-value | | p-value | [95% Conf | | Interval] | | Sig |
| --- | --- | --- | --- | --- | --- | --- | --- | --- | --- | --- | --- |
| HDL-C (mmol/L) | .444 | | .268 | -1.34 | | .179 | .136 | | 1.452 | |  |
| Constant | .395 | | .307 | -1.19 | | .233 | .086 | | 1.817 | |  |
|  | | | | | | | | | | | |
| Mean dependent var | | 0.122 | | | SD dependent var | | | 0.328 | |  |  |
| Pseudo r-squared | | 0.010 | | | Number of obs | | | 270 | |  |  |
| Chi-square | | 1.935 | | | Prob > chi2 | | | 0.164 | |  |  |
| Akaike crit. (AIC) | | 202.583 | | | Bayesian crit. (BIC) | | | 209.780 | |  |  |
| **** p<.01, ** p<.05, * p<.1* | | | | | | | | | | | |
|  | | | | | | | | | | | |

Logistic regression

| T2D | OR | | St.Err. | z-value | | p-value | [95% Conf | | Interval] | | Sig |
| --- | --- | --- | --- | --- | --- | --- | --- | --- | --- | --- | --- |
| FPG (mmol/L) | 2.976 | | 1.24 | 2.62 | | .009 | 1.315 | | 6.733 | | *** |
| Constant | .001 | | .001 | -3.50 | | 0 | 0 | | .038 | | *** |
|  | | | | | | | | | | | |
| Mean dependent var | | 0.122 | | | SD dependent var | | | 0.328 | |  |  |
| Pseudo r-squared | | 0.035 | | | Number of obs | | | 270 | |  |  |
| Chi-square | | 6.969 | | | Prob > chi2 | | | 0.008 | |  |  |
| Akaike crit. (AIC) | | 197.549 | | | Bayesian crit. (BIC) | | | 204.745 | |  |  |
| **** p<.01, ** p<.05, * p<.1* | | | | | | | | | | | |
|  | | | | | | | | | | | |

Logistic regression

| T2D | OR | | St.Err. | z-value | | p-value | [95% Conf | | Interval] | | Sig |
| --- | --- | --- | --- | --- | --- | --- | --- | --- | --- | --- | --- |
| Triglycerides (mmol/L) | 1.51 | | .348 | 1.79 | | .074 | .961 | | 2.373 | | * |
| Constant | .08 | | .03 | -6.66 | | 0 | .038 | | .168 | | *** |
|  | | | | | | | | | | | |
| Mean dependent var | | 0.122 | | | SD dependent var | | | 0.328 | |  |  |
| Pseudo r-squared | | 0.015 | | | Number of obs | | | 270 | |  |  |
| Chi-square | | 2.985 | | | Prob > chi2 | | | 0.084 | |  |  |
| Akaike crit. (AIC) | | 201.533 | | | Bayesian crit. (BIC) | | | 208.729 | |  |  |
| **** p<.01, ** p<.05, * p<.1* | | | | | | | | | | | |
|  | | | | | | | | | | | |

Logistic regression

| T2D | OR | | St.Err. | z-value | | p-value | [95% Conf | | Interval] | | Sig |
| --- | --- | --- | --- | --- | --- | --- | --- | --- | --- | --- | --- |
| Waist circumference (cm) | 1.04 | | .018 | 2.32 | | .02 | 1.006 | | 1.076 | | ** |
| Constant | .004 | | .006 | -3.51 | | 0 | 0 | | .086 | | *** |
|  | | | | | | | | | | | |
| Mean dependent var | | 0.122 | | | SD dependent var | | | 0.328 | |  |  |
| Pseudo r-squared | | 0.026 | | | Number of obs | | | 270 | |  |  |
| Chi-square | | 5.272 | | | Prob > chi2 | | | 0.022 | |  |  |
| Akaike crit. (AIC) | | 199.246 | | | Bayesian crit. (BIC) | | | 206.442 | |  |  |
| **** p<.01, ** p<.05, * p<.1* | | | | | | | | | | | |
|  | | | | | | | | | | | |

**Multivariable analyses**

Logistic regression

| T2D | OR | | St.Err. | z-value | | p-value | [95% Conf | | Interval] | | Sig |
| --- | --- | --- | --- | --- | --- | --- | --- | --- | --- | --- | --- |
| Blood pressure (mmHg) | 1.009 | | .014 | 0.70 | | .487 | .983 | | 1.037 | |  |
| Age (years) | .989 | | .029 | -0.39 | | .697 | .933 | | 1.047 | |  |
| Sex (male) | 1.547 | | .602 | 1.12 | | .262 | .722 | | 3.315 | |  |
| Education (high) | .694 | | .365 | -0.70 | | .487 | .248 | | 1.943 | |  |
| Sport (>2x/week) | 1.167 | | .454 | 0.40 | | .693 | .544 | | 2.503 | |  |
| BMI (kg/m^2^) | 1.109 | | .048 | 2.39 | | .017 | 1.019 | | 1.207 | | ** |
| Smoking | 1.188 | | .597 | 0.34 | | .733 | .443 | | 3.183 | |  |
| Positive family history of T2D | 3.552 | | 2.707 | 1.66 | | .096 | .798 | | 15.816 | | * |
| Constant | .001 | | .002 | -3.09 | | .002 | 0 | | .081 | | *** |
|  | | | | | | | | | | | |
| Mean dependent var | | 0.122 | | | SD dependent var | | | 0.328 | |  |  |
| Pseudo r-squared | | 0.065 | | | Number of obs | | | 270 | |  |  |
| Chi-square | | 13.012 | | | Prob > chi2 | | | 0.111 | |  |  |
| Akaike crit. (AIC) | | 205.506 | | | Bayesian crit. (BIC) | | | 237.892 | |  |  |
| **** p<.01, ** p<.05, * p<.1* | | | | | | | | | | | |
|  | | | | | | | | | | | |

Logistic regression

| T2D | OR | | St.Err. | z-value | | p-value | [95% Conf | | Interval] | | Sig |
| --- | --- | --- | --- | --- | --- | --- | --- | --- | --- | --- | --- |
| HDL-C (mmol/L) | .652 | | .475 | -0.59 | | .558 | .156 | | 2.722 | |  |
| Age (years) | 1.001 | | .03 | 0.03 | | .974 | .945 | | 1.061 | |  |
| Sex (male) | 1.379 | | .611 | 0.73 | | .468 | .579 | | 3.286 | |  |
| Education (high) | .687 | | .36 | -0.72 | | .474 | .246 | | 1.92 | |  |
| Sport (>2x/week) | 1.201 | | .472 | 0.46 | | .642 | .555 | | 2.596 | |  |
| BMI (kg/m^2^) | 1.109 | | .048 | 2.39 | | .017 | 1.019 | | 1.208 | | ** |
| Smoking | 1.192 | | .599 | 0.35 | | .728 | .445 | | 3.194 | |  |
| Positive family history of T2D | 3.66 | | 2.79 | 1.70 | | .089 | .822 | | 16.307 | | * |
| Constant | .004 | | .008 | -2.75 | | .006 | 0 | | .201 | | *** |
|  | | | | | | | | | | | |
| Mean dependent var | | 0.122 | | | SD dependent var | | | 0.328 | |  |  |
| Pseudo r-squared | | 0.064 | | | Number of obs | | | 270 | |  |  |
| Chi-square | | 12.883 | | | Prob > chi2 | | | 0.116 | |  |  |
| Akaike crit. (AIC) | | 205.635 | | | Bayesian crit. (BIC) | | | 238.021 | |  |  |
| **** p<.01, ** p<.05, * p<.1* | | | | | | | | | | | |
|  | | | | | | | | | | | |

Logistic regression

| T2D | OR | | St.Err. | z-value | | p-value | [95% Conf | | Interval] | | Sig |
| --- | --- | --- | --- | --- | --- | --- | --- | --- | --- | --- | --- |
| FPG (mmol/L) | 2.916 | | 1.345 | 2.32 | | .02 | 1.181 | | 7.199 | | ** |
| Age (years) | .975 | | .03 | -0.84 | | .399 | .919 | | 1.034 | |  |
| Sex (male) | 1.319 | | .532 | 0.69 | | .492 | .598 | | 2.908 | |  |
| Education (high) | .624 | | .335 | -0.88 | | .379 | .218 | | 1.787 | |  |
| Sport (>2x/week) | 1.125 | | .445 | 0.30 | | .766 | .518 | | 2.444 | |  |
| BMI (kg/m^2^) | 1.108 | | .048 | 2.37 | | .018 | 1.018 | | 1.206 | | ** |
| Smoking | 1.019 | | .522 | 0.04 | | .97 | .374 | | 2.78 | |  |
| Positive family history of T2D | 3.557 | | 2.722 | 1.66 | | .097 | .794 | | 15.937 | | * |
| Constant | 0 | | 0 | -3.84 | | 0 | 0 | | .007 | | *** |
|  | | | | | | | | | | | |
| Mean dependent var | | 0.122 | | | SD dependent var | | | 0.328 | |  |  |
| Pseudo r-squared | | 0.090 | | | Number of obs | | | 270 | |  |  |
| Chi-square | | 18.071 | | | Prob > chi2 | | | 0.021 | |  |  |
| Akaike crit. (AIC) | | 200.446 | | | Bayesian crit. (BIC) | | | 232.832 | |  |  |
| **** p<.01, ** p<.05, * p<.1* | | | | | | | | | | | |
|  | | | | | | | | | | | |

Logistic regression

| T2D | OR | | St.Err. | z-value | | p-value | [95% Conf | | Interval] | | Sig |
| --- | --- | --- | --- | --- | --- | --- | --- | --- | --- | --- | --- |
| Triglycerides (mmol/L) | 1.315 | | .35 | 1.03 | | .305 | .78 | | 2.216 | |  |
| Age (years) | .996 | | .028 | -0.13 | | .9 | .943 | | 1.053 | |  |
| Sex (male) | 1.357 | | .562 | 0.74 | | .461 | .603 | | 3.057 | |  |
| Education (high) | .695 | | .365 | -0.69 | | .488 | .248 | | 1.945 | |  |
| Sport (>2x/week) | 1.144 | | .447 | 0.35 | | .73 | .533 | | 2.459 | |  |
| BMI (kg/m^2^) | 1.106 | | .048 | 2.32 | | .02 | 1.016 | | 1.205 | | ** |
| Smoking | 1.105 | | .56 | 0.20 | | .844 | .409 | | 2.985 | |  |
| Positive family history of T2D | 3.665 | | 2.789 | 1.71 | | .088 | .825 | | 16.287 | | * |
| Constant | .002 | | .004 | -3.25 | | .001 | 0 | | .085 | | *** |
|  | | | | | | | | | | | |
| Mean dependent var | | 0.122 | | | SD dependent var | | | 0.328 | |  |  |
| Pseudo r-squared | | 0.067 | | | Number of obs | | | 270 | |  |  |
| Chi-square | | 13.532 | | | Prob > chi2 | | | 0.095 | |  |  |
| Akaike crit. (AIC) | | 204.985 | | | Bayesian crit. (BIC) | | | 237.371 | |  |  |
| **** p<.01, ** p<.05, * p<.1* | | | | | | | | | | | |
|  | | | | | | | | | | | |

Logistic regression

| T2D | OR | | St.Err. | z-value | | p-value | [95% Conf | | Interval] | | Sig |
| --- | --- | --- | --- | --- | --- | --- | --- | --- | --- | --- | --- |
| Waist circumference (cm) | .991 | | .036 | -0.24 | | .812 | .923 | | 1.064 | |  |
| Age (years) | .995 | | .028 | -0.17 | | .867 | .943 | | 1.051 | |  |
| Sex (male) | 1.69 | | .839 | 1.06 | | .29 | .639 | | 4.473 | |  |
| Education (high) | .675 | | .353 | -0.75 | | .452 | .242 | | 1.883 | |  |
| Sport (>2x/week) | 1.147 | | .45 | 0.35 | | .726 | .532 | | 2.473 | |  |
| BMI (kg/m^2^) | 1.133 | | .094 | 1.50 | | .135 | .962 | | 1.334 | |  |
| Smoking | 1.172 | | .588 | 0.32 | | .752 | .439 | | 3.132 | |  |
| Positive family history of T2D | 3.701 | | 2.817 | 1.72 | | .086 | .833 | | 16.449 | | * |
| Constant | .003 | | .007 | -2.59 | | .01 | 0 | | .245 | | *** |
|  | | | | | | | | | | | |
| Mean dependent var | | 0.122 | | | SD dependent var | | | 0.328 | |  |  |
| Pseudo r-squared | | 0.063 | | | Number of obs | | | 270 | |  |  |
| Chi-square | | 12.586 | | | Prob > chi2 | | | 0.127 | |  |  |
| Akaike crit. (AIC) | | 205.932 | | | Bayesian crit. (BIC) | | | 238.318 | |  |  |
| **** p<.01, ** p<.05, * p<.1* | | | | | | | | | | | |
|  | | | | | | | | | | | |

**MetS - effect modification by sex**

**Bivariate (main effects and interaction)**

Logistic regression

| T2D | OR | | St.Err. | z-value | | p-value | [95% Conf | | Interval] | | Sig |
| --- | --- | --- | --- | --- | --- | --- | --- | --- | --- | --- | --- |
| MetS (IDF) | 3.902 | | 2.051 | 2.59 | | .01 | 1.393 | | 10.935 | | *** |
| Sex (male) | 1.494 | | .808 | 0.74 | | .458 | .518 | | 4.311 | |  |
| **MetS * Sex** | **.738** | | **.563** | **-0.40** | | **.691** | **.166** | | **3.286** | |  |
| Constant | .072 | | .026 | -7.18 | | 0 | .035 | | .148 | | *** |
|  | | | | | | | | | | | |
| Mean dependent var | | 0.122 | | | SD dependent var | | | 0.328 | |  |  |
| Pseudo r-squared | | 0.056 | | | Number of obs | | | 270 | |  |  |
| Chi-square | | 11.187 | | | Prob > chi2 | | | 0.011 | |  |  |
| Akaike crit. (AIC) | | 197.331 | | | Bayesian crit. (BIC) | | | 211.724 | |  |  |
| **** p<.01, ** p<.05, * p<.1* | | | | | | | | | | | |

**Multivariable**

Logistic regression

| T2D | OR | | St.Err. | z-value | | p-value | [95% Conf | | Interval] | | Sig |
| --- | --- | --- | --- | --- | --- | --- | --- | --- | --- | --- | --- |
| MetS (IDF) | 2.893 | | 1.595 | 1.93 | | .054 | .982 | | 8.521 | | * |
| Sex (male) | 1.544 | | .848 | 0.79 | | .429 | .526 | | 4.529 | |  |
| **MetS * Sex** | **.882** | | **.697** | **-0.16** | | **.873** | **.187** | | **4.154** | |  |
| Age (years) | 1 | | .029 | 0.02 | | .987 | .945 | | 1.059 | |  |
| Education (high) | .649 | | .345 | -0.81 | | .416 | .229 | | 1.841 | |  |
| Sport (>2x/week) | 1.26 | | .509 | 0.57 | | .568 | .571 | | 2.78 | |  |
| BMI (kg/m^2^) | 1.079 | | .05 | 1.65 | | .1 | .986 | | 1.18 | | * |
| Smoking | .991 | | .515 | -0.02 | | .986 | .358 | | 2.743 | |  |
| Positive family history of T2D | 3.447 | | 2.64 | 1.62 | | .106 | .768 | | 15.463 | |  |
| Constant | .003 | | .006 | -2.93 | | .003 | 0 | | .15 | | *** |
|  | | | | | | | | | | | |
| Mean dependent var | | 0.122 | | | SD dependent var | | | 0.328 | |  |  |
| Pseudo r-squared | | 0.092 | | | Number of obs | | | 270 | |  |  |
| Chi-square | | 18.541 | | | Prob > chi2 | | | 0.029 | |  |  |
| Akaike crit. (AIC) | | 201.977 | | | Bayesian crit. (BIC) | | | 237.962 | |  |  |
| **** p<.01, ** p<.05, * p<.1* | | | | | | | | | | | |

**MetS - effect modification by family history of T2D**

**Bivariate (main effects and interaction)**

Logistic regression

| T2D | OR | | St.Err. | z-value | | p-value | [95% Conf | | Interval] | | Sig |
| --- | --- | --- | --- | --- | --- | --- | --- | --- | --- | --- | --- |
| MetS (IDF) | 0 | | .001 | -0.01 | | .994 | 0 | | . | |  |
| Positive family history of T2D | 1.672 | | 1.307 | 0.66 | | .511 | .361 | | 7.743 | |  |
| **MetS * FH_T2D** | **8015743.2** | | **1.562e+10** | **0.01** | | **.993** | **0** | | **.** | |  |
| Constant | .056 | | .04 | -3.98 | | 0 | .013 | | .231 | | *** |
|  | | | | | | | | | | | |
| Mean dependent var | | 0.122 | | | SD dependent var | | | 0.328 | |  |  |
| Pseudo r-squared | | 0.083 | | | Number of obs | | | 270 | |  |  |
| Chi-square | | 16.675 | | | Prob > chi2 | | | 0.001 | |  |  |
| Akaike crit. (AIC) | | 191.843 | | | Bayesian crit. (BIC) | | | 206.237 | |  |  |
| **** p<.01, ** p<.05, * p<.1* | | | | | | | | | | | |

**Multivariable**

Logistic regression

| T2D | OR | | St.Err. | z-value | | p-value | [95% Conf | | Interval] | | Sig |
| --- | --- | --- | --- | --- | --- | --- | --- | --- | --- | --- | --- |
| MetS (IDF) | 0 | | .001 | -0.01 | | .989 | 0 | | . | |  |
| Positive family history of T2D | 1.519 | | 1.204 | 0.53 | | .598 | .321 | | 7.179 | |  |
| **MetS * FH_T2D** | **3012581.9** | | **3.093e+09** | **0.01** | | **.988** | **0** | | **.** | |  |
| Sex (male) | 1.491 | | .592 | 1.01 | | .314 | .685 | | 3.248 | |  |
| Age (years) | 1.003 | | .029 | 0.09 | | .925 | .947 | | 1.061 | |  |
| Education (high) | .61 | | .325 | -0.93 | | .354 | .215 | | 1.734 | |  |
| Sport (>2x/week) | 1.235 | | .494 | 0.53 | | .598 | .564 | | 2.706 | |  |
| BMI (kg/m^2^) | 1.084 | | .05 | 1.75 | | .08 | .99 | | 1.186 | | * |
| Smoking | .966 | | .502 | -0.07 | | .947 | .349 | | 2.675 | |  |
| Constant | .006 | | .011 | -2.72 | | .007 | 0 | | .235 | | *** |
|  | | | | | | | | | | | |
| Mean dependent var | | 0.122 | | | SD dependent var | | | 0.328 | |  |  |
| Pseudo r-squared | | 0.108 | | | Number of obs | | | 270 | |  |  |
| Chi-square | | 21.656 | | | Prob > chi2 | | | 0.010 | |  |  |
| Akaike crit. (AIC) | | 198.861 | | | Bayesian crit. (BIC) | | | 234.846 | |  |  |
| **** p<.01, ** p<.05, * p<.1* | | | | | | | | | | | |
