# Supplementary material for: A 14-year prospective cohort study of type 2 diabetes development in Dutch healthy adults of South Asian origin: risk factors and the association with metabolic syndrome and HOMA-IR
Source: Acta Diabetol. 2025 May 12;62(11):1873–80. doi: 10.1007/s00592-025-02513-3 (PMC12640338; doi:10.1007/s00592-025-02513-3)
Supplement: Supplementary file 4 — Supplementary Data 4 (DOCX 19 KB) [file 592_2025_2513_MOESM4_ESM.docx]

**Supplemental Data 4 – Baseline Characteristics**

Supplementary Table 1

| Characteristic | Total (N=698) | Non-Completers (N=428) | Completers (N=270) | p-value |
| --- | --- | --- | --- | --- |
| Sex – female | 402 (58%) | 242 (57%) | 160 (59%) | 0.48 |
| Age | 45 (8) | 46 (8) | 44 (7) | 0.041 |
| Metabolic Syndrome (IDF) | 215 (31%) | 127 (30%) |  |  |
| Education |  |  |  |  |
| Low/Middle | 537 (77%) | 323 (75%) | 214 (79%) | 0.31 |
| High | 159 (23%) | 103 (24%) | 56 (21%) |  |
| Missing | 2 (0%) | 0 (0%) | 0 (0%) |  |
| Smoking | 135 (19%) | 88 (21%) | 47 (17%) | 0.30 |
| Positive family history of T2D | 573 (82%) | 351 (82%) | 222 (82%) | 0.95 |
| Sport < 3 days per week | 400 (57%) | 236 (55%) | 164 (61%) | 0.15 |
| Systolic blood pressure (mmHg) | 133 (16) | 133 (16) | 132 (15) | 0.92 |
| Diastolic blood pressure (mmHg) | 85 (10) | 85 (10) | 85 (9) | 0.62 |
| HbA1c (mmol/mol) | 38 (x/1) | 38 (x/1) | 38 (x/1) | 0.81 |
| LDL-C (mmol/L) | 3.4 (0.8) | 3.4 (0.8) | 3.4 (0.8) | 0.56 |
| HDL-C (mmol/L) | 1.3 (0.3) | 1.3 (0.3) | 1.3 (0.3) | 0.78 |
| Triglycerides (mmol/L) | 1.1 (x/1.7) | 1.1 (x/1.7) | 1.1 (x/1.7) | 0.47 |
| Insulin (mIU/L) | 10.2 (x/2.3) | 10.1 (x/2.3) | 10.4 (x/2.2) | 0.69 |
| Glucose (mmol/L) | 4.9 (x/1.1) | 5.0 (x/1.1) | 4.9 (x/1.1) | 0.043 |
| Waist circumference (cm) | 88 (x/1) | 88 (x/1) | 89 (x/1) | 0.44 |
| W/H ratio | 0.89 (0.07) | 0.89 (0.07) | 0.88 (0.07) | 0.50 |
| BMI (kg/m^2^) | 26.1 (3.9) | 25.9 (3.9) | 26.4 (4.0) | 0.10 |

Supplementary Table 2

| Characteristic | Total (N=270) | No Metabolic Syndrome (N=182) | Metabolic Syndrome (N=88) | p-value |
| --- | --- | --- | --- | --- |
| Sex – female | 160 (59%) | 117 (64%) | 43 (49%) | 0.016 |
| Age | 44 (7) | 44 (7) | 44 (6) | 0.85 |
| T2D | 33 (12%) | 14 (8%) | 19 (22%) | 0.001 |
| Education |  |  |  |  |
| Low/Middle | 214 (79%) | 143 (79%) | 71 (81%) | 0.69 |
| High | 56 (21%) | 39 (21%) | 17 (19%) |  |
| Smoking | 47 (17%) | 27 (15%) | 20 (23%) | 0.11 |
| Positive family history of T2D | 222 (82%) | 145 (80%) | 77 (88%) | 0.11 |
| Sport < 3 days per week | 164 (61%) | 103 (57%) | 61 (69%) | 0.045 |
| Systolic blood pressure (mmHg) | 132 (15) | 130 (15) | 139 (13) | <0.001 |
| Diastolic blood pressure (mmHg) | 85 (9) | 83 (9) | 89 (9) | < 0.001 |
| HbA1c (mmol/mol) | 38 (x/1) | 37 (x/1) | 39 (x/1) | 0.002 |
| LDL-C (mmol/L) | 3.4 (0.8) | 3.4 (0.8) | 3.5 (0.8) | 0.51 |
| HDL-C (mmol/L) | 1.3 (0.3) | 1.4 (0.3) | 1.1 (0.2) | < 0.001 |
| Triglycerides (mmol/L) | 1.1 (x/1.7) | 0.9 (x/1.6) | 1.6 (x/1.6) | < 0.001 |
| Insulin (mIU/L) | 10.4 (x/2.2) | 8.7 (x/2.3) | 14.8 (x/2.1) | < 0.001 |
| Glucose (mmol/L) | 4.9 (x/1.1) | 5.0 (x/1.1) | 5.0 (x/1.1) | 0.001 |
| Waist circumference (cm) | 89 (x/1) | 86 (x/1) | 94 (x/1) | < 0.001 |
| W/H ratio | 0.88 (0.07) | 0.87 (0.07) | 0.91 (0.07) | < 0.001 |
| BMI (kg/m^2^) | 26.4 (3.9) | 25.5 (3.9) | 28.1 (3.7) | < 0.001 |
